# Amelioration of Paracetamol-Induced Hepatotoxicity in Rat by the Administration of Methanol Extract of *Muntingia calabura* L. Leaves

**DOI:** 10.1155/2014/695678

**Published:** 2014-04-24

**Authors:** N. D. Mahmood, S. S. Mamat, F. H. Kamisan, F. Yahya, M. F. F. Kamarolzaman, N. Nasir, N. Mohtarrudin, S. F. Md. Tohid, Z. A. Zakaria

**Affiliations:** ^1^Department of Biomedical Sciences, Faculty of Medicine and Health Sciences, Universiti Putra Malaysia (UPM), 43400 Serdang, Selangor, Malaysia; ^2^Department of Pathology, Faculty of Medicine and Health Sciences, Universiti Putra Malaysia, 43400 Serdang, Selangor, Malaysia; ^3^Integrative Pharmacogenomic Institute, Universiti Teknologi MARA, Level 7, FF3 Building, 42300 Bandar Puncak Alam, Selangor, Malaysia

## Abstract

*Muntingia calabura* L. is a tropical plant species that belongs to the Elaeocarpaceae family. The present study is aimed at determining the hepatoprotective activity of methanol extract of *M. calabura* leaves (MEMC) using two models of liver injury in rats. Rats were divided into five groups (*n* = 6) and received 10% DMSO (negative control), 50 mg/kg N-acetylcysteine (NAC; positive control), or MEMC (50, 250, and 500 mg/kg) orally once daily for 7 days and on the 8th day were subjected to the hepatotoxic induction using paracetamol (PCM). The blood and liver tissues were collected and subjected to biochemical and microscopical analysis. The extract was also subjected to antioxidant study using the 2,2-diphenyl-1-picrylhydrazyl-(DPPH) and superoxide anion-radical scavenging assays. At the same time, oxygen radical antioxidant capacity (ORAC) and total phenolic content were also determined. From the histological observation, lymphocyte infiltration and marked necrosis were observed in PCM-treated groups (negative control), whereas maintenance of hepatic structure was observed in group pretreated with N-acetylcysteine and MEMC. Hepatotoxic rats pretreated with NAC or MEMC exhibited significant decrease (*P* < 0.05) in ALT and AST enzymes level. Moreover, the extract also exhibited good antioxidant activity. In conclusion, MEMC exerts potential hepatoprotective activity that could be partly attributed to its antioxidant activity and, thus warrants further investigations.

## 1. Introduction

Liver is a vital organ that plays a role in controlling critical biochemical and physiological activities including homeostasis, growth, energy and nutrient supply, detoxification of drugs and other xenobiotics, and also combating infections [1, 2]. Therefore, it is very susceptible to being damaged by hepatotoxic agents [3]. Many newly developed drugs (e.g., rimonabant, propylthiouracil, or corticosteroids) have been used for treatment of liver diseases; however, these drugs possess harmful side effects such as insomnia, vomiting, constipation, and depression. For that reason, further research on plants and herbs that could potentially substitute the chemical-based drugs is very crucial as many medicinal plants have been found to possess hepatoprotective properties [4]. One of the plants that are currently being investigated for its potential pharmacological activities in our laboratory is* Muntingia calabura, *or locally known as “ceri kampung.” Traditionally, the Peruvian folklore believed that* M. calabura* leaf can reduce gastric ulcer and swelling of prostate gland and alleviate headache and cold [5]. Scientifically, it has been proven that the leaves possess various pharmacologic activities, including antiulcer [6], antinociceptive, antipyretic, and anti-inflammatory activities [7]. An* in vitro* study had demonstrated that* M. calabura* possessed antioxidant and antiproliferative activities [8]. From our literature review, no attempt has been made to study the hepatoprotective potential of* M. calabura *leaves. Various reports had shown that the antioxidant and anti-inflammatory activities played significant role in the mechanisms of hepatoprotective activity [9, 10]. Therefore, in accordance with those reports and the fact that* M. calabura *also exerted antioxidant and anti-inflammatory activities as discussed above, the hypothesis that the extract of* M. calabura *will also demonstrate hepatoprotective potential possibly via the same antioxidant and anti-inflammatory mechanisms is worth justifying. Therefore, the present study was aimed at determining the hepatoprotective activity of methanol extract of* M. calabura* leaves (MEMC) using the paracetamol- (PCM-) induced liver damage in rats as the animal model.

## 2. Materials and Methods

### 2.1. Chemicals

Paracetamol (PCM;* Sigma*-Aldrich, USA) and N-acetylcysteine (NAC; Acros Organics, USA) were used in the study. All other chemicals and reagents used were of analytical grade.

### 2.2. Collection of Plant Material

The leaves of* M. calabura *were collected around Universiti Putra Malaysia (UPM), Serdang campus, Selangor, Malaysia, which were then identified by comparison with specimens available at the Herbarium of the Laboratory of Natural Products, IBS, UPM, Serdang, Selangor, Malaysia. A voucher specimen (SK 2198/13) has been issued. The leaves were dried under shade for 7 days at room temperature, separated, and pulverized by mechanical grinder to form coarse powder.

### 2.3. Preparation of Plant Extract

The coarse powder of the air-dried leaves of* M. calabura* was subjected to methanol extraction whereby 1 kg of powder leaves was macerated in 20 L of methanol in the ratio of 1 : 20 (w/v) for 72 hours. The supernatant was filtered sequentially using cloth filter, cotton wool, and Whatman filter paper number. 1. The solvent was then evaporated under reduced pressure (204 mbar) and controlled temperature (40°C) using a vacuum rotary evaporator (Buchi Rotavapor R210/215, Switzerland). The whole processes were repeated twice for the remaining residue [11].

### 2.4. Animals

Healthy male Sprague Dawley rats at 8-9 weeks of age weighing 180–220 g were used throughout the study. Animals were obtained from the Animal House Facility, Faculty of Medicine and Health Sciences, Universiti Putra Malaysia. They were housed at room temperature of 27−30°C and allowed free access to food and tap water* ad libitum*. The animals were acclimatized to laboratory conditions for 7 days before the commencement of experiments. The study protocol of the present study was approved by the Animal House and Use Committee, Faculty of Medicine and Health Sciences, UPM (ethical approval number: UPM/FPSK/PADS/BR-UUH/00449). The rats were handled in accordance with current UPM guidelines for the care of laboratory animals and the ethical guidelines for investigations of experimental pain in conscious animals. All experiments were conducted between 09.30 and 18.30 h to minimize the effects of environmental changes.

### 2.5. Pharmacological Studies

#### 2.5.1. Antioxidant Activity of MEMC

In an attempt to measure the antioxidant activity, the DPPH free radical scavenging assay was carried out according to the procedure described by Blois [12] with slight modification. Initially, the sample serial dilution was performed to obtain final concentrations of 200, 100, 50, 25, 12.5, 6.25, and 3.13 *μ*g/mL solutions from 1.0 mg/mL stock sample. Next, in 96-well microtiter plate, 50 *μ*L of the previously prepared solutions was added to 50 *μ*L of DPPH (FG: 384.32) (1 mM in ethanolic solution) and 150 *μ*L of ethanol (absolute) in triplicates. The plate was shaken (15 seconds, 500 rpm) and left to stand at room temperature for 30 minutes. The absorbance of the resulting solution was measured spectrophotometrically at 520 nm. Different concentrations of L-ascorbic acid (3.13–200 *μ*g/mL) were used as the standard antioxidant. The control was prepared by adding 50 *μ*L deionized water to 950 *μ*L 100 *μ*M DPPH reagent and the analysis was followed as described above. The results were expressed as percentage inhibition (*I*%) using the following equation:

(1) $$\begin{array}{c} I\%=\frac{\left({\text{Abs}}_{\text{control}}-{\text{Abs}}_{\text{sample}}\right)}{{\text{Abs}}_{\text{control}}}\times 100. \end{array}$$

Abs_control_ is the absorbance of the control reaction with 50 *μ*L deionized water without the extract or ascorbic acid, and Abs_sample_ is the absorbance in the presence of the sample. The effective concentration of the sample required to scavenge DPPH radical by 50% (EC_50_) was obtained by linear regression analysis of dose response curve plotting between* I*% and concentrations.

### 2.6. Hepatoprotective Assay

The* in vivo* hepatoprotective activity of MEMC was determined using the PCM-induced hepatotoxicity test in rats. The animals (*n* = 6) were randomly divided into 6 experimental groups and administered with test solutions as follows.

- Group I serving as normal control received 10% DMSO.
- Group II serving as negative control received 10% DMSO.
- Group III serving as positive control received 50 mg/kg NAC.
- Pretreatment groups:
  - group IV received 50 mg/kg MEMC,
  - group V received 250 mg/kg MEMC,
  - group VI received 500 mg/kg MEMC.

These doses of extract (50, 250, and 500 mg/kg) were used in the present study based on previous report on the acute toxicity study performed using three doses (300, 500, and a maximum dose of 2000 mg/kg MEMC) administered orally, which showed no signs of toxicity in rats [13].

The animals were fasted for 48 hours prior to the experiment under standard laboratory conditions but allowed free access to distilled water (dH_2_O)* ad libitum*. After 48 hours, each group received the respective dose of test solution orally once daily for 7 consecutive days. The oral administration of PCM was performed 3 hours after the last extract administration on the 7th day except for group I, which received only 10% DMSO. After 48 hours of hepatic injury induction, the animals were lightly anesthetized using diethyl ether and the blood was collected by cardiac puncture in sterilized centrifuged tubes which was then centrifuged at 3000 rpm for 10 minutes to get serum for biochemical parameters study. The animals were then sacrificed by cervical dislocation and the liver was removed for histopathological studies.

### 2.7. Liver Enzymes Assessment

Serum collected was assayed according to the standard liver enzymes assessment methods. Alanine aminotransferase (ALT), alkaline phosphate (ALP), and aspartate aminotransferase (AST) levels were measured using the Hitachi 902 Automatic Chemical Analyser.

### 2.8. Histopathology

The liver tissue was dissected out and fixed in the 10% formalin, dehydrated in gradual ethanol (50–100%), cleared in xylene, and embedded in paraffin wax. The sections, which were 5-6 mm thick, were then prepared using rotary microtome (Leica RM 2125 RTS, Singapore) and stained with hematoxylin and eosin dye for microscopic observation of histopathological changes in the liver. Next, the liver sections were scored and evaluated according to the severity of the hepatic injury as described by El-Beshbishy et al. [14] with slight modifications.

### 2.9. Phytochemical Screening and HPLC Analysis of MEMC

The phytochemical screening of dried leaves of MEMC was performed according to the standard screening tests and conventional protocols as adopted by Zakaria et al. [7]. The HPLC analysis of MEMC was performed according to the method of Zakaria et al. [7] with slight modifications. Briefly, 10 mg of MEMC was dissolved in 1 mL MeOH and then filtered through the filterer membrane with the pore size of 0.45 µm. The filtered MEMC was then analyzed using a Waters Delta 600 with 600 Controller and Waters 2996 Photodiode Array (Milford, MA, USA), which was equipped with an autosampler, online degasser and column heater. Data was evaluated and processed using the installed Millenium 32 Software (Waters Product). The filtered MEBP was separated on a minibore Phenomenex Luna 5 mm C_18_ column (dimensions 250 × 4.60 mm) at 27°C using a one-step linear gradient. The sample was eluted using the solvent system consisting of 0.1% aqueous formic acid (labelled as solvent A) and acetonitrile (labelled as solvent B) and two types of elution systems were used as follows: (i) Initial conditions were 85% A and 15% B with a linear gradient reaching 25% B at *t* = 12 min. This was maintained for 10 min after which the programmed returned to the initial solvent composition at *t* = 25 min and continued for 10 min. (ii) Initial conditions were 95% A and 5% B with a linear gradient reaching 25% B at *t* = 12 min. This was maintained for 10 min after which the gradient was reduced to 15% B at *t* = 22 min and maintained for another 8 min (*t* = 30 min). The programme was returned to the initial solvent composition at *t* = 35 min. The flow rate used was 1.0 mL/min and the injection volume was 10 µL. The HPLC was monitored at 254 and 366 nm. Further analysis was also carried out to compare the HPLC chromatogram of MEMC against several pure compounds of flavonoid types (e.g. fisetin, quercetin, rutin, quercitrin, naringenin, genistein, pinostrobin, hesperetin and flavanone).

### 2.10. Statistical Analysis

Data obtained are presented as mean ± standard error of mean (SEM). The data were analysed using one-way analysis of variance (ANOVA) and the differences between the groups were determined using Dunnet post hoc test with *P* < 0.05 as the limit of significance.

## 3. Results

### 3.1. Antioxidant Studies of MEMC

**Figure 1:**
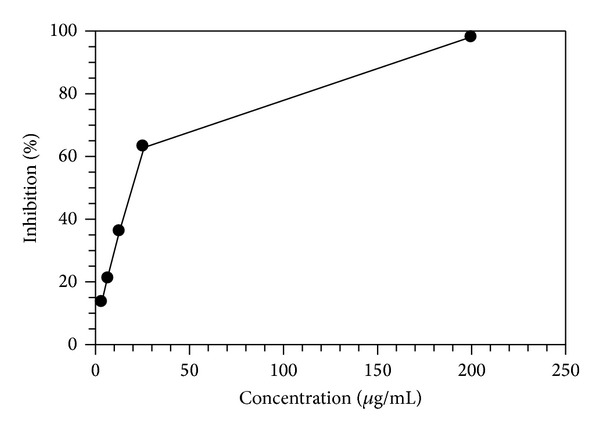
Antioxidant activity of MEMC measured using the* in vitro* DPPH assay.

Scavenging of DPPH represents the free radicals reducing activity of antioxidants based on a one-electron reduction which was determined by the decrease of its absorbance at 520 nm. The MEMC exhibited significant antioxidant activity in the DPPH assay in a concentration-dependent manner, as illustrated in Figure 1. The IC_50_ value obtained was 17.39 ± 0.74 *μ*g/mL which is comparable to the reference standard green tea extract (13.90 *μ*g/mL).

### 3.2. *In Vivo* Hepatoprotective Study

#### 3.2.1. Effect of MEMC on the Body Weight, Liver Weight, and Liver Weight/Body Weight (LW/BW Ratio) after Induction with PCM

The administration of PCM following pretreatment with 10% DMSO (negative group) did not significantly (*P* < 0.05) cause increase in the average body weight when compared to the normal control group. The MEMC, at 250 and 500 mg/kg, and 50 mg/kg NAC treated groups showed a significant (*P* < 0.05) decrease in the average body weight when compared to the negative control group (Table 1).

On the other hand, PCM administration did cause significant (*P* < 0.05) increase in the average liver weight of group pretreated with 10% DMSO when compared to the normal control group. However, only pretreatment with 500 mg/kg MEMC caused significant (*P* < 0.05) reduction in the average liver weight of rats induced with PCM. The 50 mg/kg NAC failed to reduce the increase in liver weight when compared to the negative control group (Table 1).

The mean relative liver weights (LW/BW ratio) of acute PCM-treated animals (negative control) showed significant increase compared to the control normal group (*P* < 0.05). Only the PCM-treated group that was pretreated with 500 mg/kg MEMC showed a significant (*P* < 0.05) decrease in the value of the mean relative liver weights (Table 1).

#### 3.2.2. Histopathological Study of the PCM-Induced Liver Toxicity with and without Pretreatment of MEMC

**Figure 2:**
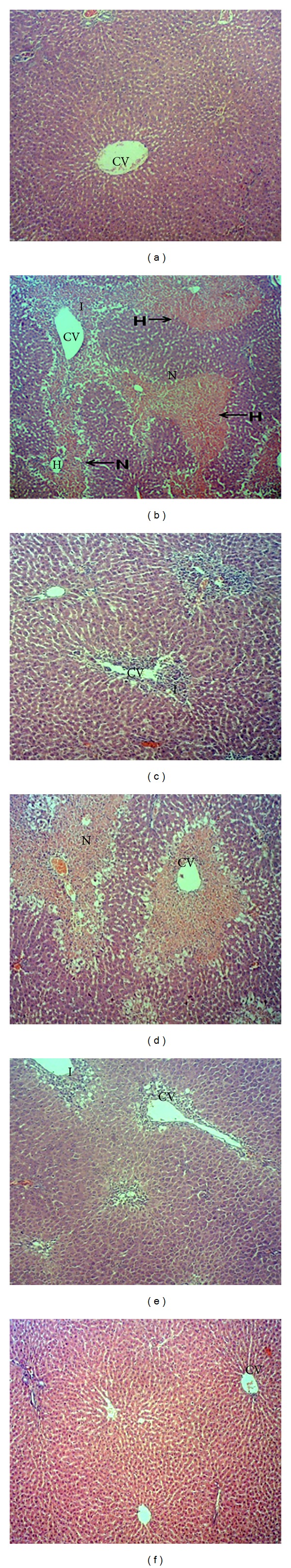
Microscopic observations of liver tissue pretreated with various concentrations of MEMC followed by treatment against PCM-induced liver injury: (a) normal, (b) section of liver tissue of 3 g/kg PCM-treated group (p.o.) showing massive necrosis, haemorrhage, and inflammation, (c) section of 50 mg/kg of N-acetylcysteine liver tissue pretreated on the liver followed by PCM showing preservation of normal hepatocytes, (d) section of pretreated 50 mg/kg MEMC liver tissue followed by PCM showing tissue necrosis and inflammation, (e) section of pretreated 250 mg/kg MEMC liver tissue followed by PCM showing mild inflammation, and (f) section of pretreated 500 mg/kg MEMC liver tissue followed by PCM showing normal histology with mild inflammation (40x magnification). CV: central vein; N: necrosis; I: inflammation; H: haemorrhage.

Histopathological observations (Table 2) performed in this study demonstrated that the normal control group (non-PCM-intoxicated liver pretreated with 10% DMSO) showed normal lobular architecture and normal hepatic cells with intact cytoplasm and well-defined sinusoids (Figure 2(a)). The section of PCM intoxicated liver, pretreated with 10% DMSO (Figure 2(b)), exhibited massive necrosis, presence of haemorrhage, and inflammation with infiltration of lymphocytes involving mainly centrilobular zone 3. Interestingly, these pathological changes were found to be reduced with the increasing doses of MEMC indicating the extract ability to reverse the PCM-induced intoxication (Figures 2(d)–2(f)).  Table 3 shows the histopathological scoring of the liver tissues pretreated with the respective test solution. The presence of marked necrosis, hemorrhage, and inflammation following treatment with PCM (showed by the negative control group) had reduced remarkably when pretreated with MEMC or NAC.

#### 3.2.3. Effects of MEMC on Liver Enzymes

**Figure 3:**
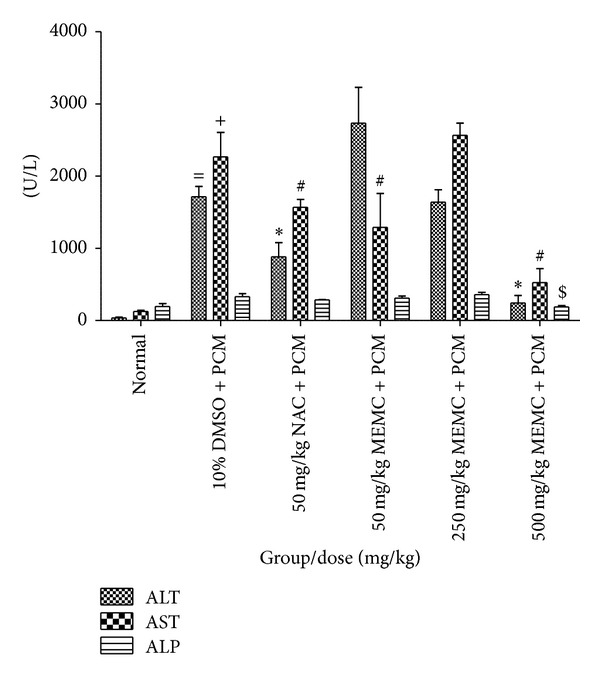
Effect of various doses of MEMC on the serum ALT, AST, and ALP (U/L) levels assessed against PCM-induced hepatic injury in rats. ^=^Significantly different (*P* < 0.05) as compared to the ALT level in the normal control group. ^+^Significantly different as compared to the AST level in the normal control group. *Significantly different as compared to the ALT level in the 10% DMSO + PCM-treated group. ^#^Significantly different as compared to the AST level in the 10% DMSO + PCM-treated group. ^$^Significantly different as compared to the ALP level in the 10% DMSO + PCM-treated group.

In this study, significant elevations of ALT, AST, and ALP were recorded in negative control group as compared to the normal, non-PCM intoxicated group (Table 3, Figure 3). In addition, the histopathological study of the PCM-intoxicated liver pretreated with the respective test solution exhibited correlation with serum biochemical indices. Interestingly, the oral administration of 500 mg/kg MEMC and 50 mg/kg NAC exhibited significant reduction on the level of these enzymes.

### 3.3. Phytochemical Constituents and HPLC Profile of MEMC

Phytochemical investigation on the crude extract revealed the presence of various compounds, such as flavonoids, tannins, polyphenols, saponins and steroids and the absence of triterpenes and alkaloids.

**Figure 4:**
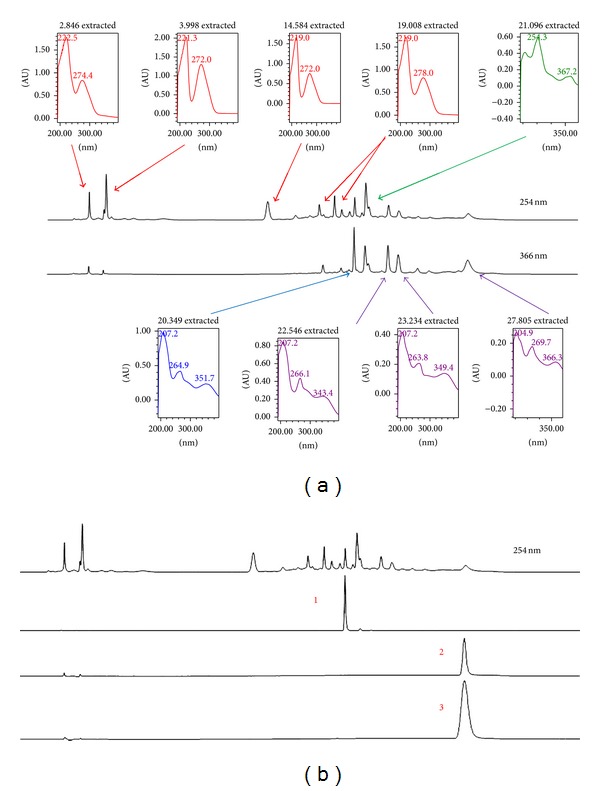
HPLC profile of MEMC. (a) HPLC chromatogram of MEMC at 254 nm and 366 nm. Approximately eleven major peaks were detected at 254 nm with some of them being further highlighted at 366 nm. Each peak was represented by their respective UV-Vis spectra with *λ*_max⁡_ value. (b) HPLC chromatogram of MEMC at 254 nm compared against several standard pure flavonoids demonstrated the presence of, namely, rutin (1), fisetin (2), and quercetin (3).

The HPLC analysis of MEMC was measured at the wavelength of 254 nm and revealed nine major peaks, which were P1 (RT = 2.846 min), P2 (RT = 3.998 min), P3 (RT = 14.584 min), P4 (RT = 19.008 min), P5 (RT = 21.096 min), P6 (RT = 20.349 min), P7 (RT = 22.546 min), P8 (RT = 23.234 min), and P9 (RT = 27.805 min) (Figure 4(a)). Comparison between chromatogram of the standard compounds with chromatogram of MEMC revealed the presence of rutin, quercetin, and fisetin (Figure 4(b)).

## 4. Discussion and Conclusion

PCM, an over-the-counter drug, is a commonly used antipyretic and analgesic which can lead to liver damage if taken in overdose [15, 16]. In therapeutic dose, PCM is converted by drug metabolizing enzymes to water-soluble metabolites and secreted in the urine [17, 18]. Saturated and excess PCM is oxidatively metabolized by hepatic cytochrome p450 (CYP450) system to a toxic metabolite, namely,* N*-acetyl-*p*-benzoquinone imine (NAPQI) [19–21]. The NAPQI is normally detoxified by a nonprotein thiol known as glutathione (GSH) with both oxidant scavenger and redox-regulation capacities [20]. GSH is a major antioxidant system and a crucial component of host defense which is responsible for scavenging reactive free radicals produced through the metabolism process within the liver to prevent cell injury [16, 22]. The toxic dose of PCM caused the depletion of GSH resulting in accumulation of NAPQI which then covalently binds to the cysteinyl sulfhydryl groups of cellular proteins forming NAPQI-protein adducts [23, 24]. This event results in the generation of reactive oxygen species (ROS) including the hydrogen peroxide (H_2_O_2_), superoxide anion (O_2_^−^), and hydroxyl (OH^−^) radical that affect the cellular membrane and induce lipid peroxidation and also cause hepatic necrosis [15, 20]. The hepatic cell injuries cause the leaking of cellular enzymes into the blood stream and thus can be measured in the serum [16]. The ALT is an essential serum biomarker of liver damage [25] along with the AST and ALP that are routinely assessed to monitor the function status of the liver [26].

The PCM-induced toxicity model is commonly used to study the potential hepatoprotective activity of extracts/compounds [16, 27]. In the present investigation, the 3 g/kg of PCM which is a toxic dose, has resulted in the increment of body weight, liver weight, and LW/BW ratios of rats and showed significant elevation of serum level of hepatic enzymes ALT, AST, and ALP in comparison to normal control group, as expected. Interestingly, administration of MEMC successfully lowered the level of these enzymes and concurrently showed the capability to reduce the liver weight and LW/BW ratios of rats in a dose-independent manner. The failure of PCM to affect body weight in this acute model of hepatotoxicity was parallel with report made by Saad et al. [28] on the failure of thioacetamide to cause changes in body weight of rats in acute liver injury study. Despite the significant changes in liver weights as well as liver body weight ratios observed in PCM-treated rats compared to rats in control groups, measurement of liver body weight ratio is a more accurate approach to determine the changes in liver size compared to the measurement of liver weight alone as the liver weightlargely depends on the size of the rat. The enlargement of livers in PCM-treated rats suggested hepatic lesions and liver injury associated with the toxic effects of PCM. These significant changes in the liver weights may be attributed to the accumulation of extracellular matrix protein and collagen in liver tissue.

The role of PCM reactive metabolite NAPQI as described previously is responsible for the development of PCM-induced hepatotoxicity which seems to depend partly on the existence of free radicals and oxidative processes. Therefore, it is hypothesized that extracts/compounds with free radical scavenging and/or antioxidant activities could also exhibit hepatoprotective activity against the PCM-induced liver toxicity. Interestingly, this is supported by the study of Gupta et al. [29] that claimed that the combination of hepatoprotective effect and antioxidant activity synergistically prevents the process of initiation and progression of hepatocellular injury [29]. Besides that, previous findings demonstrated the MEMC ability to scavenge free radicals and to exhibit antioxidant activity [30], which is concurrent with our recent study using the DPPH assay. Furthermore, the inflammatory process has also been thought to exacerbate chemical-induced hepatotoxicity. For example, PCM intoxication triggers the release of various mediators that are involved in the production of reactive oxygen species and nitric oxide that can affect liver damage or repair [31]. Therefore, it is possible to propose that the extract/compound exerting an anti-inflammatory activity might also demonstrate hepatoprotective activity. In addition, the leaves of* M. calabura* have also been previously reported to exhibit anti-inflammatory activity [7, 32]. In the present study, MEMC exerted hepatoprotective activity against PCM-induced liver damage in a dose-dependent manner as suggested by the microscopic analysis. Although MEMC did not show a dose-dependent activity against the serum liver enzymes level, the highest dose of MEMC exhibited a significant decrease in serum liver enzymes level. The finding is further supported by the normalization of histopathological changes to preserve the histostructure of hepatocytes. In addition, the MEMC-induced hepatoprotective effects were almost comparable to the standard hepatoprotective drug, NAC.

Phytochemical screening of MEMC demonstrated the presence of flavonoids, saponins, and tannins, as well as the existence of phenolic compounds as indicated by high total phenolic content (TPC) value [8]. The hepatoprotective potential of MEMC can be explained based on the respective phytoconstituents detected in the extract. For example, flavonoids have been reported to exert antioxidant [33, 34], anti-inflammatory [35], and hepatoprotective [34, 35] activities. Moreover, saponins have been reported to exert hepatoprotective activity via modulation of its antioxidant [36] and anti-inflammatory activities [37], while condensed tannins have been suggested to possess free radical scavenging and antioxidant, anti-inflammatory and hepatoprotective activities [38]. Based on all of the reports, the MEMC-induced hepatoprotective activity is suggested to possibly involve the synergistic actions of flavonoids, saponins, and condensed tannins. The HPLC analysis of MEMC demonstrated the presence of at least eleven major fractions with some of the peaks detected at the UV-Vis spectra with *λ*_max⁡_ value, which falls within the range that detected flavonoids [39]. Moreover, some of those detected peaks have been demonstrated to represent rutin, fisetin, and quercetin.

Detail studies on the phytochemical constituents of* M. calabura *leaves, in particular, demonstrated the presence of various types of flavonoid-based compounds [40–44]. Interestingly, the isolation and identification of those flavonoid-based bioactive compounds were carried out on the leaves part extracted using methanol, which is similar to the MEMC used in the present study. The presence of flavonoids in MEMC was also expected based on the HPLC analysis wherein some of the peaks detected in the UV spectra represent flavonoid-based compounds. It is important to highlight that flavonoids can be divided into five major subgroups, namely, flavonols, flavones, dihydroflavonols, flavanonols, and flavanones [39]. The UV-Vis spectra of flavonoids consist of two absorbance bands labeled as A and B. Band A falls in the range of 310–350 nm for flavones and 350–385 nm for flavonols while Band B falls in the range of 250–290 nm and is similar for all of the abovementioned subgroups. As for the dihydroflavonols and flavanones, the wavelength of Band A falls within the range of 300–330 nm while Band B lies within the range of 277–295 nm. Other than those facts, flavonols and various polyphenols have been shown to exert maximal absorbance at variable wavelengths between 270 and 290 nm. Flavonoids, in particular, have been reported to possess hepatoprotective properties and could be the one responsible for the observed MEMC activity.

Interestingly, the ability of MEMC to exert hepatoprotective activity possibly via its antioxidant action is in line with our previous report on the hepatoprotective activity of methanol extract of* Bauhinia purpurea* (MEBP) [16]. Comparison was made between the HPLC chromatogram of MEMC and MEBP (chromatogram not shown) and, interestingly, rutin, quercetin, and fisetin were detected in MEMC while gallic acid and catechin were detected in MEBP. Despite almost similar mechanisms of hepatoprotection, both extracts contained different types of phytoconstituents. These differences will provide advantage in improving the antioxidant activity and, concomitantly, the hepatoprotective activity of those plants/extracts if they are combined and tested together due, possibly, to the synergistic effect of various compounds.

MEMC had successfully reversed the PCM-induced hepatotoxic effect by its ability to reduce the elevated level of ALT, AST, and ALP suggesting that these biochemical restorations could be due to the extract ability to inhibit the cytochrome P450 or/and ability to promote the PCM glucuronidation [45]. Furthermore, the ability to lower the enzymes level can be associated with the ability of MEMC to prevent lipid peroxidation of endoplasmic reticulum that is rich in polyunsaturated fatty acid by disrupting the binding of activated radicals to the macromolecules. This process can possibly be achieved via the antioxidant activity of MEMC [46]. Besides, mechanisms of protection that can take place include activation of liver regeneration by enhancing the protein and glycoprotein synthesis or accelerated detoxification and excretion [47], prevention of lipid peroxidation process, and stabilization of hepatocellular membrane [46]. However, the results obtained warrant further studies, and more detailed investigations are currently underway to determine the possible hepatoprotective mechanism(s) involved and to isolate and identify the responsible bioactive compounds derived from MEMC.

## Figures and Tables

**Table 1:** Effect of MEMC on percentage change of body and liver weight in PCM-induced hepatic injury rats.

Treatment	Dose (mg/kg)	Body weight, BW (g)	Liver weight, LW (g)	LW/BW (%)
Normal	—	207.9 ± 4.741	6.190 ± 0.3565	2.967 ± 0.1097
10% DMSO + PCM	—	217.5 ± 8.258	8.797 ± 0.7331^a^	4.023 ± 0.2399^a^
NAC + PCM	50	189.9 ± 2.697^a^	8.232 ± 0.3992^a^	4.326 ± 0.1522^a^
MEMC + PCM	50	193.3 ± 9.105^b^	8.009 ± 0.5417^a^	4.136 ± 0.1568^a^
	250	195.9 ± 1.893^ab^	8.844 ± 0.1816^a^	4.513 ± 0.0718^a^
	500	176.7 ± 3.130^ab^	6.340 ± 0.4192^b^	3.583 ± 0.2096^b^

Values are expressed as means ± SEM of six replicates.

^a^Significantly different as compared to normal control group, *P* < 0.05. ^b^Significantly different as compared to negative control group, *P* < 0.05.

**Table 2:** Histopathological scoring of the tissue of PCM-induced hepatic injury rats after pretreatment with MEMC.

Treatment	Dose (mg/kg)	Steatosis	Necrosis	Inflammation	Haemorrhage
Normal	−	−	−	−	−
10% DMSO + PCM		−	+++	++	++
NAC + PCM	50	−	+	+	−
MEMC + PCM	50	−	++	+	+
	250	−	+	+	−
	500	−	+	+	−

The severity of various features of hepatic injury was evaluated based on those following scoring schemes: − normal, + mild effect, ++ moderate effect, and +++ severe effect.

**Table 3:** Effect of MEMC on the ALT, AST, and ALP (U/L) level following its pretreatment against the PCM-induced hepatic injury.

Treatment	Dose (mg/kg)	ALT (U/L)	AST (U/L)	ALP (U/L)
Normal	—	36.05 ± 10.52	124.3 ± 16.14	193.0 ± 41.44
10% DMSO + PCM		1714 ± 142.2^#^	2266 ± 340.4^#^	330.0 ± 42.35^#^
NAC + PCM	50	884.2 ± 195.4*	1569 ± 106.4*	284.3 ± 5.536*
MEMC + PCM	50	2734 ± 495.2*	1292 ± 468.0*	311.5 ± 25.64
	250	1638 ± 174.4	2565 ± 170.5	359.0 ± 32.73
	500	244.9 ± 101.9*	526.1 ± 191.1*	221.7 ± 25.55*

Values are expressed as means ± SEM of six replicates.

^#^Significantly different as compared to normal group, *P* < 0.05.

*Significantly different as compared to negative control (10% DMSO + PCM), *P* < 0.05.
